# COP29: incremental but insufficient progress on climate finance

**DOI:** 10.1016/j.lanepe.2024.101169

**Published:** 2024-12-06

**Authors:** The Lancet Regional Health – Europe

At COP29 in Baku, Azerbaijan, which was extended until Nov 24, 2024, climate finance took centre stage. Dubbed the “finance COP,” the conference's conclusion was delayed as nearly 200 countries struggled to agree on the New Collective Quantified Goal (NCQG)—a climate funding plan for the next decade to replace the US$100 billion annual target expiring in 2025. The NCQG is seen as crucial to achieving the Paris Agreement's aim of limiting global warming to 1.5 °C.

After extended negotiations and walkouts from small island and least-developed nations, historic emitters pledged $300 billion annually by 2035 for climate finance—far below the $500 billion per year demanded by developing countries. The agreement also set a broader target of $1.3 trillion per year by 2035, combining public and private funding. Although UN-commissioned climate experts agree that $1.3 trillion is the minimum needed for these nations to tackle the climate crisis, some estimates suggest that up to $7 trillion annually is necessary to account for the Global North's historical responsibility for emissions.

The NCQG agreement at COP29 represents yet another missed opportunity for meaningful climate justice. Although it introduces a mix of grants and low-interest loans, the continued reliance on loans exacerbates the debt burdens of vulnerable nations already reeling from escalating climate impacts. Calls for a shift to predominantly grant-based financing—essential to equity—were largely ignored, leaving developing countries to bear disproportionate costs for a crisis they did not cause.

Another landmark decision was made to fully operationalise the Loss and Damage Fund by 2025. This long-awaited milestone builds on commitments from COP27 and COP28 to support vulnerable nations, including small island states, least-developed countries, and African nations, in addressing climate impacts. Although pledges exceeding $730 million to date are a step forward, the amount remains disappointingly low and falls short of the $100 billion proposed by the countries bearing the brunt of climate impacts.

Despite setbacks at COP29, the EU has reaffirmed its leadership in climate action, presenting its first Biennial Transparency Report, highlighting progress on support for developing nations to reduce greenhouse gas emissions, while also outlining future climate finance plans. As the largest contributor to international climate finance, the EU provided over €28.6 billion in 2023, with plans to increase financing in line with the NCQG. The EU has also committed to reducing emissions by 90% by 2040, tripling renewable energy capacity, and doubling energy efficiency by 2030. Additionally, the EU co-launched a statement on gender-responsive climate action, highlighting the disproportionate impact of climate change on women and their crucial role in decision making.

However, at COP29, not only did the financial commitments fall short of expectations, but the event also highlighted two serious issues: first, conflicts of interest, with fossil fuel lobbyists exerting significant influence on negotiations, and the continued dominance of the fossil fuel industry in pushing their deals. Over 1700 fossil fuel lobbyists, many linked to national delegations, gained access to the talks, raising serious concerns about their influence on climate policy. This access is particularly disappointing and contrary to the purpose of COP, especially given the pledges at COP28 to phase out fossil fuel subsidies. Investment in new fossil fuel supply surpassed $1 trillion last year, and emissions from coal, oil, and gas are expected to rise by 0.8% in 2024—far from the 43% reduction needed by 2030 to meet the 1.5 °C target.

Second, geopolitical challenges, including the absence of key leaders from major polluting nations like the USA, China, and India, reflected a concerning lack of political will for binding commitments on climate finance. The return of Donald Trump to the US presidency and his promise to withdraw the USA, the world's largest historical emitter, from the Paris Agreement add further uncertainty about the continuity of US climate commitments. Additionally, the fact that only eight of 78 Heads of State at COP29 were women underscores the conference's ongoing representation issues.

Despite the incremental progress made at COP29, the urgency of the climate crisis demands more than promises and financial agreements—it requires bold, actionable support for vulnerable nations. Global temperatures in 2024 have already surged 1.54 °C above pre-industrial levels, and annual climate-related losses are set to escalate beyond the $143 billion annually recorded from 2000 to 2019. Current financial pledges fall woefully short of addressing the escalating scale of climate disasters. At COP30 in Belém, Brazil, in 2025, countries must prioritise transformative, equitable solutions and counter the influence of fossil fuel lobbyists. As a global leader, the EU must step up with robust climate finance commitments, particularly if US leadership wanes.

